# AI-assisted Segmentation Tool for Brain Tumor MR Image Analysis

**DOI:** 10.1007/s10278-024-01187-7

**Published:** 2024-07-08

**Authors:** Myungeun Lee, Jong Hyo Kim, Wookjin Choi, Ki Hong Lee

**Affiliations:** 1 Research Institute of Medical Sciences, Chonnam National University, Gwangju, Republic of Korea; 2 Department of Cardiovascular Medicine, Chonnam National University Hospital, Gwangju, Republic of Korea; 3 Department of Radiology, Seoul National University Hospital, Seoul, Republic of Korea; 4 Department of Applied Bioengineering, Graduate School of Convergence Science and Technology, Seoul National University, Suwon, Republic of Korea; 5 Department of Radiation Oncology, Sidney Kimmel Medical College, Thomas Jefferson University, Philadelphia, PA USA; 6 Department of Internal Medicine, Chonnam National University Medical School, Gwangju, Republic of Korea

**Keywords:** Segmentation, Semi-Automated, Magnetic Resonance Imaging, Brain Tumor

## Abstract

TumorPrism3D software was developed to segment brain tumors with a straightforward and user-friendly graphical interface applied to two- and three-dimensional brain magnetic resonance (MR) images. The MR images of 185 patients (103 males, 82 females) with glioblastoma multiforme were downloaded from The Cancer Imaging Archive (TCIA) to test the tumor segmentation performance of this software. Regions of interest (ROIs) corresponding to contrast-enhancing lesions, necrotic portions, and non-enhancing T2 high signal intensity components were segmented for each tumor. TumorPrism3D demonstrated high accuracy in segmenting all three tumor components in cases of glioblastoma multiforme. They achieved a better Dice similarity coefficient (DSC) ranging from 0.83 to 0.91 than 3DSlicer with a DSC ranging from 0.80 to 0.84 for the accuracy of segmented tumors. Comparative analysis with the widely used 3DSlicer software revealed TumorPrism3D to be approximately 37.4% faster in the segmentation process from initial contour drawing to final segmentation mask determination. The semi-automated nature of TumorPrism3D facilitates reproducible tumor segmentation at a rapid pace, offering the potential for quantitative analysis of tumor characteristics and artificial intelligence-assisted segmentation in brain MR imaging.

**Supplementary Information:**

The online version contains supplementary material available at 10.1007/s10278-024-01187-7.

## Introduction

Tumor segmentation is a crucial task for quantitative analysis in diverse radiological applications and yet remains a challenging problem, especially in magnetic resonance (MR) imaging due to highly heterogeneous tissue contrast in different sequences. Once correctly segmented, the shape and tissue contrast of a tumor may provide important information for radiological decision-making. In particular, accurate segmentation of brain tumors on MR images can have a considerable impact on differential diagnosis, growth rate prediction, and treatment planning [1]. However, some brain tumors such as gliomas and glioblastomas are much more difficult to delineate than other brain tumors since these tumors tend to be diffuse and poorly contrasted and thus difficult to segment.

Numerous segmentation algorithms with a broad spectrum of techniques ranging from manual slice-by-slice outline generation to fully automated segmentation have been developed. When selecting a segmentation software tool or method, the balance between efficiency and quality of segmentation should be considered [Table 1].

**Table 1:**
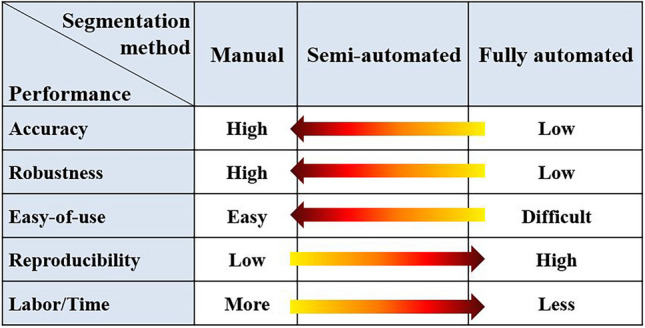
Performance attributes of image segmentation methods

Considerable research efforts have recently attempted to develop machine learning (ML)-based segmentation algorithms [1]. Clustering can be considered an unsupervised learning approach that is widely utilized for many ML applications [2–4]. The popularity of this approach is due to its ability to partition data according to certain similarity criteria. Zhang et al. [5] proposed a hybrid clustering technique combined with morphological operations for brain tumor segmentation. Supervised learning techniques employ training samples labeled by experts for learning a network. Supervised models based on ML and deep learning (DL) are currently employed in numerous computer vision applications including natural language processing [6–8] and medical image processing [9, 10]. However, despite the advances in learning-based automatic segmentation methods, many clinical studies still rely on interactive segmentation due to the limited reliability and accuracy of fully automated methods.

We published [11] a semi-automated lesion segmentation algorithm based on an active surface model that uses sketch drawings provided by the user. In particular, this algorithm can tailor key model parameters required to perform improved segmentation for heterogeneous tumor contrasts depending on organs, diseases, and imaging modalities. However, a major drawback of the work is that it requires intensive user involvement in tuning parameters. Over the last several years, insights gathered from the results of our previous segmentation model indicated that discovering a well-tuned parameter set required considerable effort and experience as well as access to a quality tumor database.

To resolve this issue, we undertook the development of an improved tumor segmentation model with a set of model parameters specifically tuned for brain tumors on MR images. Herein, we present a semi-automated tumor segmentation software tool called *TumorPrism3D*.

In this software, the entire workflow from creating and storing the tumor region of interest (ROI) mask to three-dimensional (3D) visualization is integrated in a single platform. In addition, the combination of parameters optimized for tumor segmentation is reported. *TumorPrism3D* showed highly accurate and fast tumor segmentation with a user-friendly interface.

Our contributions in this work are the following:

- Effectiveness: *TumorPrism3D* shows good performance by combining optimized parameters and providing an auto-save option for segmentation results.
- Easy to use: Minimal user intervention is required.
- Reproducibility: High and low signal portions can be treated using the optimized parameters that were tested experimentally.
- Quantitative Analysis: Our software has the potential to be used in quantitative analysis of tumor characteristics in multiparametric MR imaging.

## Materials and Methods

### Image Data

The MR images of 185 patients with glioblastoma multiforme (103 males, 82 females) were downloaded from The Cancer Imaging Archive (TCIA) database [12], in which post-contrast T1-weighted imaging and fluid-attenuated inversion recovery (FLAIR) MR sequences were tested. The downloaded data were selected based on the size of the tumors, and tumor data were too small (less than 1 mm) were excluded. In addition, the Multimodal Brain Tumor Image Segmentation Benchmark (BRATS) dataset including low/high grade, which was used at the multimodal brain tumor segmentation challenge in MICCAI 2015 [13], was tested.

ROIs corresponding to the contrast-enhancing lesions, necrotic portions, and edema components were segmented for each tumor. Tumor segmentation was performed by two experienced raters (with 17 years and 10 years of experience) for each tumor tissue component using semi-automated segmentation software tools. In addition, to evaluate the accuracy of the computer-assisted semi-automated tumor segmentation, we created a reference tumor segmentation dataset for the same cases evaluated by two radiologists (with over 20 years of experience) in consensus. Contrast-enhancing and the necrotic lesions have strong contrast compared to parenchymal tissue on the T1-weighted images. Edema components are relatively large and show ambiguous contrast and fuzzy boundary edges on FLAIR images.

### Semi-Automated Tumor Segmentation Software

3DSlicer [14], NordicICE [15], and MIM [16] are semi-automated tools. Among them, 3DSlicer (http://www.slicer.org) is the most popular open-source software and has been used for the analysis and visualization of medical images. The development of 3DSlicer—including its numerous modules, extensions, datasets, pull requests, patches, issues reports, and suggestions—is made possible by users, developers, contributors, and commercial partners around the world [14]. Among numerous modules, we focused on the image segmentation module to compare with TumorPrism3D in this study. Segment the Editor module of 3DSlicer offers a wide range of segmentation methods and has many merits. However, 3DSlicer has some limitations such as high interobserver variability according to the skill of the user; furthermore, no auto-save option exists, and the user must save everything manually. On the other hand, our software, TumorPrism3D, has a simpler segmentation process and faster execution time than the commonly used 3DSlicer software. In addition, TumorPrism3D was designed to provide robust segmentation performance for the tumors with low contrast boundaries to be more accurate and smoother on the segmented mask. Moreover, by default, TumorPrism3D saves segmented masks automatically to an output folder with the same filename as the input folder. The following will describe the technical principles and usage of TumorPrism3D.

#### Technical Principles

Our previous work [11] contains detailed equations and information about the newly proposed algorithm, but the program had to be run step by step because previous work did not integrate the segmentation process with the batch processing. Visualization and saving for the segmented masks were also separated.

In this section, we focus on the technical part to obtain accurate segmentation results and demonstrate how to use the graphical user interface (GUI).

An active surface model based on a level set method using a hybrid speed function is available within TumorPrism3D. The hybrid speed function $F$ consists of three energy terms (edge_ ${S}_{e}$, region_ ${S}_{r}$, and smoothing term_ ${S}_{s}$):

1 $$F=\alpha {S}_{e}+{\beta S}_{r}+{\gamma S}_{s}$$

where α, β, and γ are weights for edge, region, and smoothing terms, respectively.

The user can manipulate several parameters simultaneously from the control panel to find a set of values that are appropriate for a particular segmentation task. The details of these three energy terms are described in Supplementary 1.

#### Graphical Interface and Usage

TumorPrism3D is a segmentation platform written in MATLAB and designed for the tumor segmentation and visualization of brain MR images. All the functions are accessible through its GUI without MATLAB programming experience. The GUI was designed with a straightforward user interface. The multiple functions of the software are not listed in long menus; they are accessible only when needed and are typically suggested within contextual popup menus or specific interface windows. This structure provides faster and easier access to the necessary functions.

**Fig. 1:**
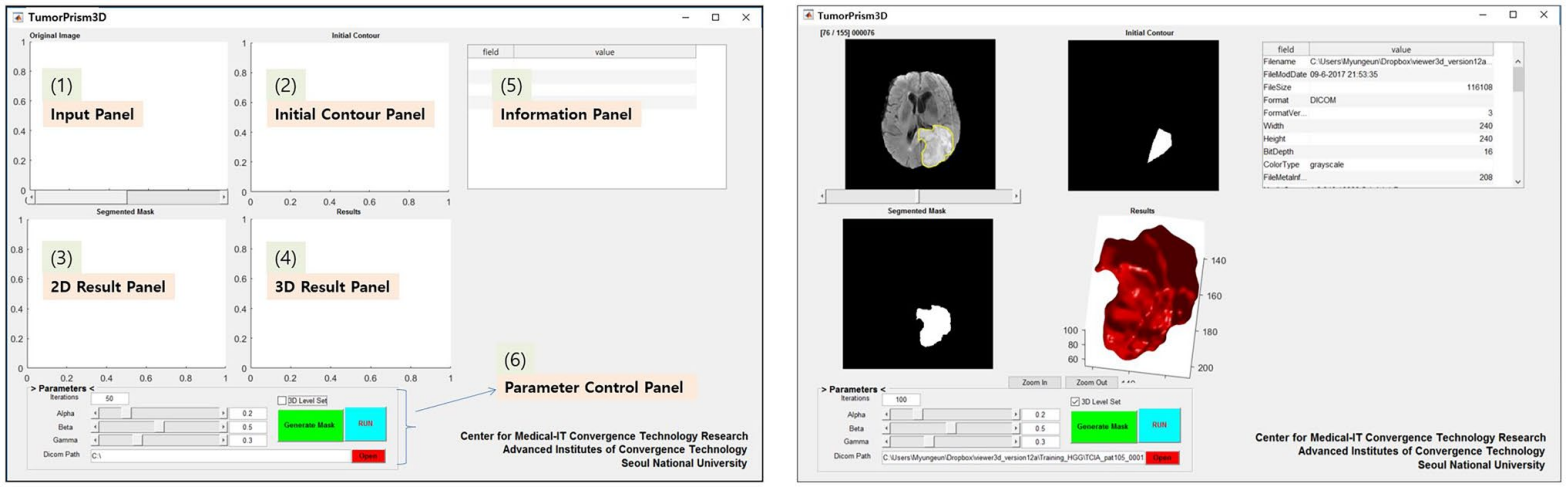
Front panel of the TumorPrism3D interface (left) and an example panel after segmenting (right)

Figure 1 shows our GUI, which consists of the following six panels:

1. **Input Panel:** Loads and shows the input DICOM image. Superimposes the initial or segmented contour to the original image. After loading the image, this panel is controlled by the slide bar at the bottom.
2. **Initial Contour Panel:** Displays the initial contour or volume drawn by the user. For the 3D image, only one slide is available to draw initial contour; then, the contour is automatically propagated to the previous and next slides.
3. **2D Result Panel:** Displays each slide of the segmented tumor mask by controlling the slide bar.
4. **3D Result Panel:** Provides interactive 3D rendering of the segmented tumor, which is available to zoom in/out.
5. **Information Panel:** Displays the image metadata from the DICOM tags of the currently active image, such as the file format, gender, age, pixel spacing, width, height, and manufacturer.
6. **Parameter Control Panel:** Provides controls for parameter setting. Through an optimal combination of three parameter values and the number of iterations, the work mode can be run. After automatic deformation, result mask files are saved in the DICOM format and are organized in a structured database folder.

The tumor segmentation procedure with TumorPrism3D is as follows:

- **(Step 1) Data loading**: Load input data; 2D a single image file / 3D – an image folder
- **(Step 2) Initial contour drawing**: (1) Left click on the image contour panel to start drawing the initial contour, (2) add boundary points, and (3) right click to finish it. 2D – Draw an initial contour within the tumor, 3D – (1) Check the ‘3D Level Set’ checkbox on the parameter control panel and (2) draw the initial contour.
- **(Step 3) Parameter setting**: Set the iteration times and three parameters on the parameter control panel. In this step, the optimal parameters will be automatically set as default, which is directly applicable to segment without any further parameter setting.
- **(Step 4) Segmentation execution:** Click the ‘RUN’ button on the parameter control panel.
- **(Step 5) Parameter tuning:** If the results are unacceptable, the user can tune the parameters (step 3) and run the segmentation process again (step 4).

**Fig. 2:**
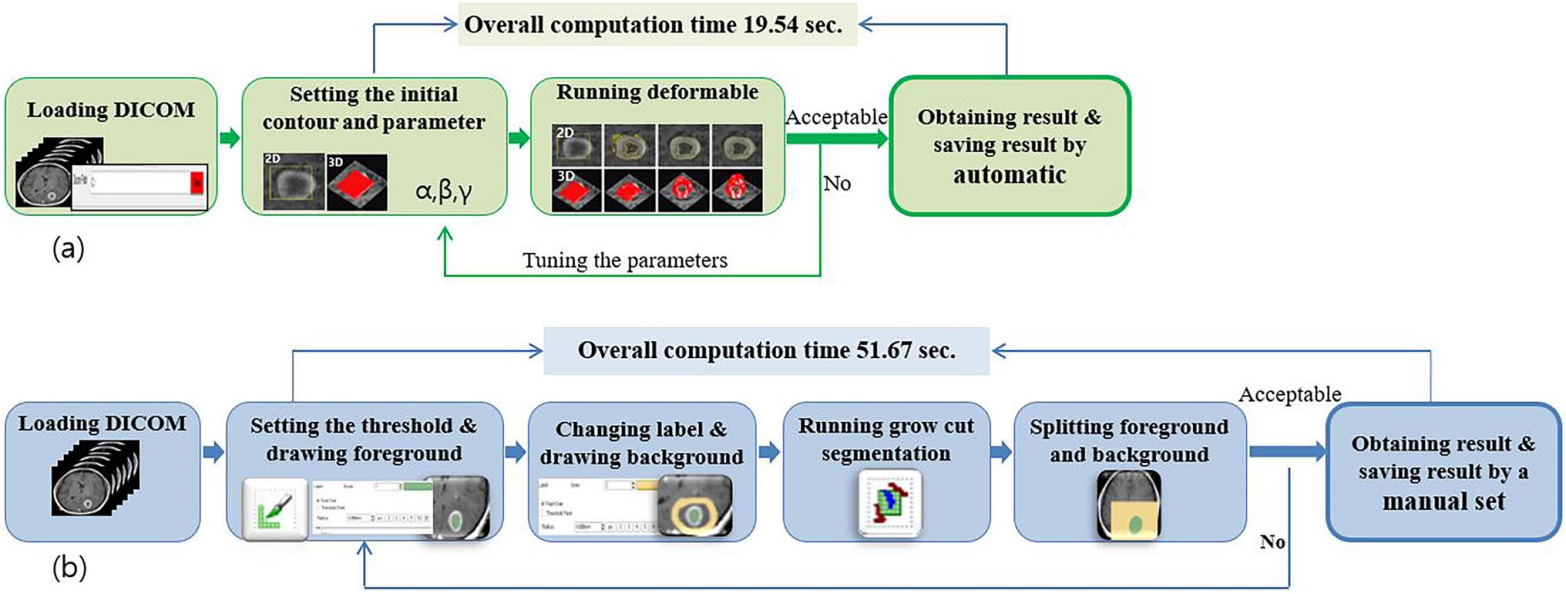
Tumor segmentation procedures: **a** TumorPrism3D and **b** 3DSlicer

Excluding parameter tuning, TumorPrism3D segments a tumor through the four steps from data loading to the segmentation execution. An auto-save option is available for each segmentation result that will be saved. The auto-save option increases usability. On the other hand, 3DSlicer has three more steps than TumorPrism3D, and the user must manually save all results after segmentation. Moreover, 3DSlicer requires the user to define background and foreground objects by drawing sketches on them, followed by the automated separation of a tumor from background tissues using the grow-cut algorithm. Figure 2 shows a comparison of tumor segmentation procedures between TumorPrism3D and 3DSlicer (ver.4.3.1).

### Tumor Segmentation Analysis

The properties of the three energy terms in the hybrid speed function were tested. The three parameters (α, β, and γ) of the terms should be set and weighted properly to guide the evolving surface under different input image conditions. In TumorPrism3D, particularly to segment the edema region with a similar intensity area, the region term needs a larger weight (β ≥ 0.5) than the other terms. Furthermore, the large weight of the smoothing term cannot maintain the boundary shape of various brain tumors; therefore, the weight factor (γ ≤ 0.3) should be defined in an appropriate range.

Next, to evaluate the accuracy of the two semi-automated tumor segmentation software programs, we created a reference tumor segmentation dataset for the same cases from in-house software data acquired by two radiologists in consensus. We used the Dice similarity coefficient (DSC) to calculate the similarity between the two segmented tumor volumes.

## Results

**Fig. 3:**
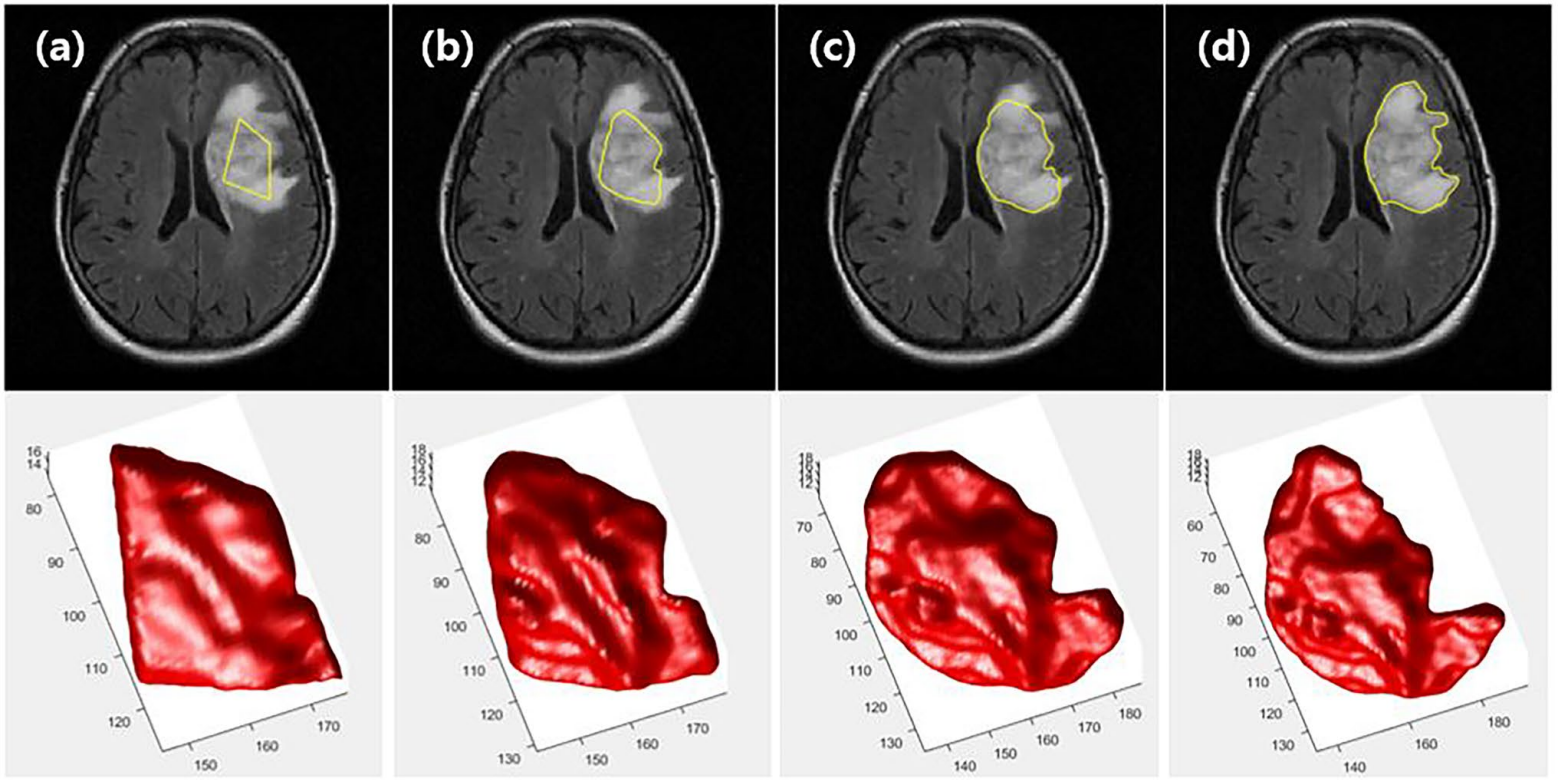
Tumor segmentation results of the TumorPrism3D: **a** The user starts drawing a contour inside the tumor, and the segmented area automatically expands. **b** Intermediate segmentation closer to the initial image. **c** Intermediate segmentation closer to the final image. **d** Final result

The tumor segmentation results of each step are shown in Fig. 3. The algorithm starts with an initial contour that was interactively drawn by the user. The initial contour area gradually expands to the final segmentation.

**Fig. 4:**
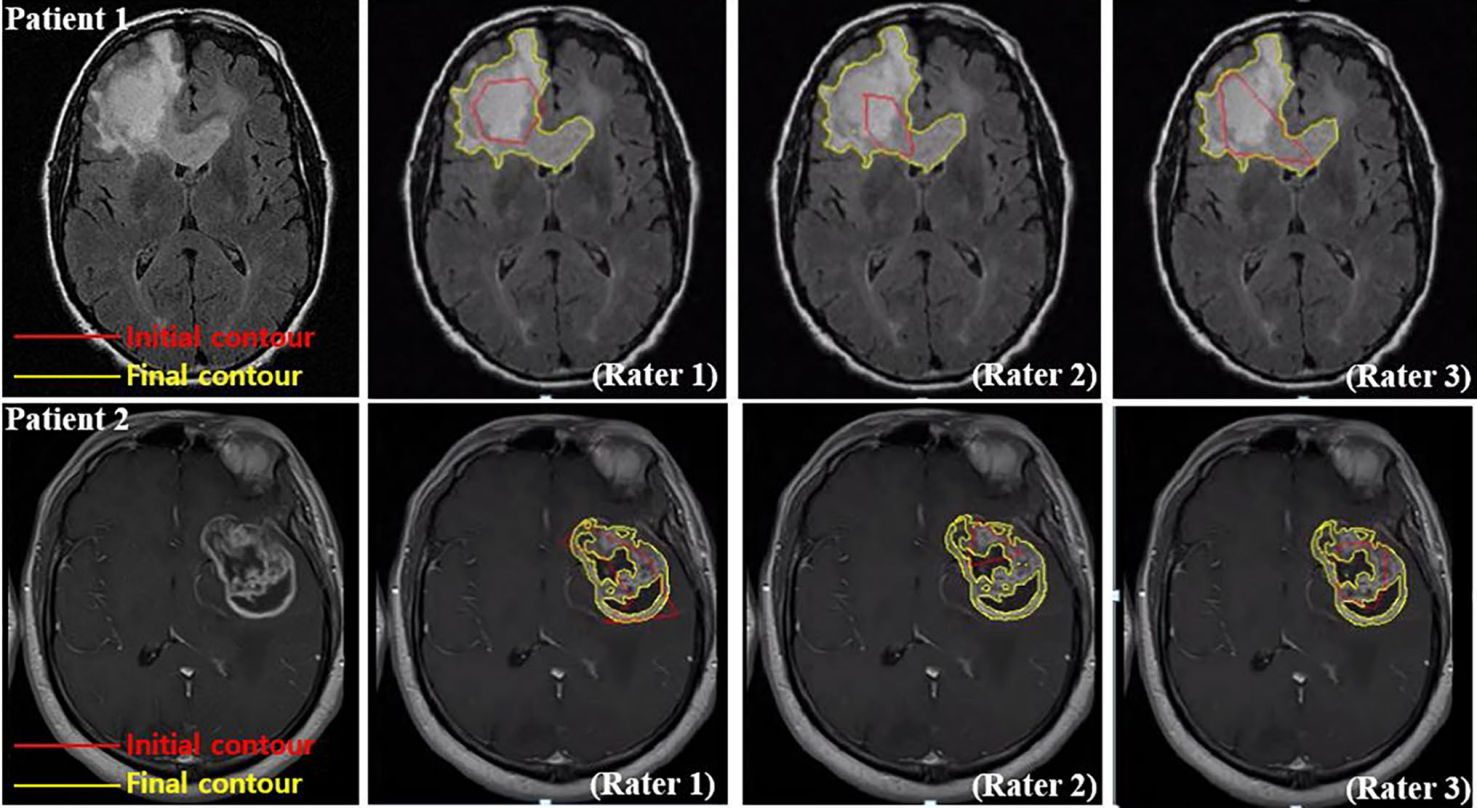
Three different initializations for applying on the reference slice

The robustness of the proposed method to the initial contour was tested using various initial contours. Figure 4 shows examples of the initial contours and the final results, demonstrating that TumorPrism3D is robust and independent of the initial contours drawn by different raters.

**Fig. 5:**
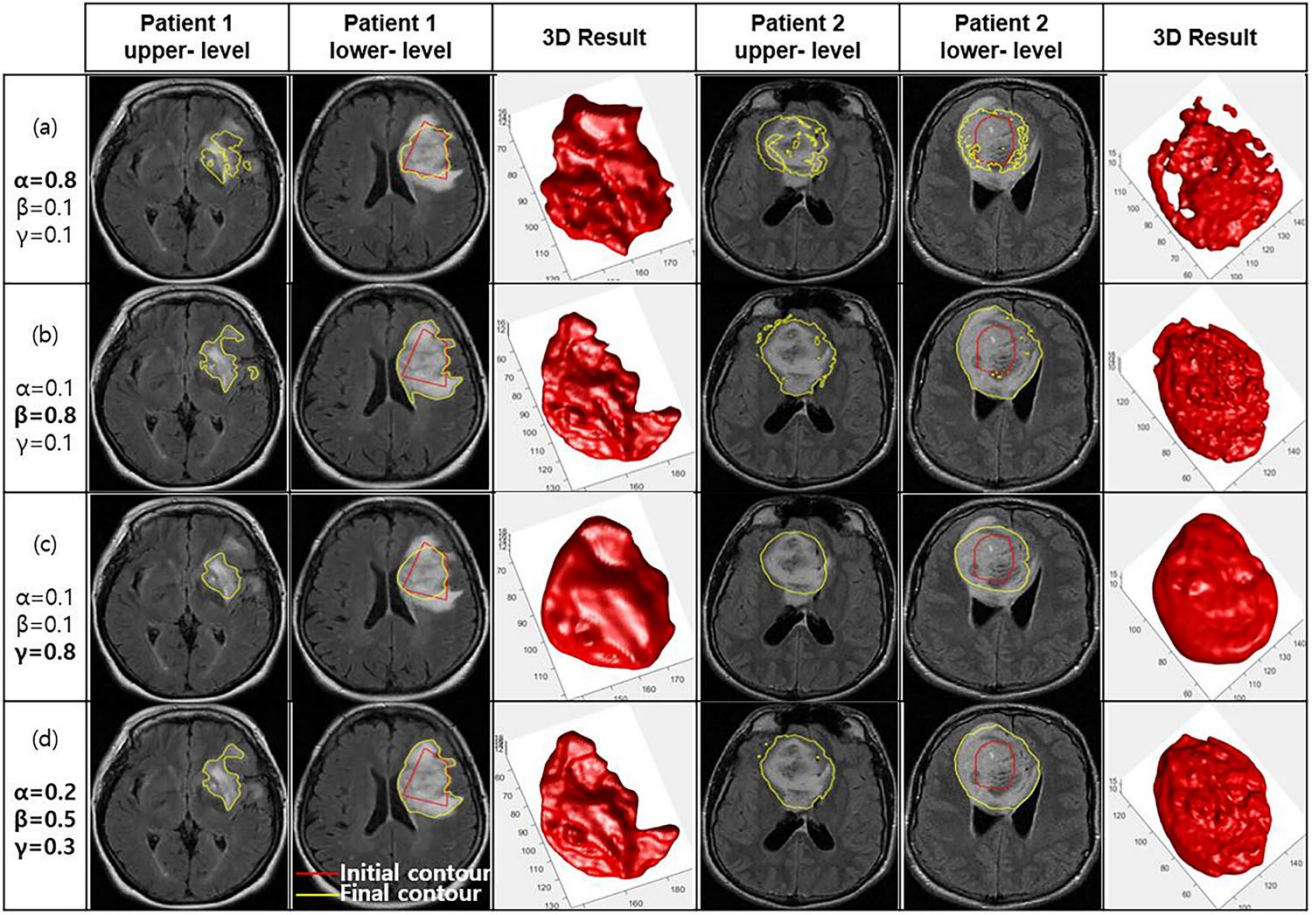
Segmentation results according to three different parameter settings: **a** with a strong edge term (α = 0.8, β = 0.1, γ = 0.1), **b** a strong region term (α = 0.1, β = 0.8, γ = 0.1), **c** a strong smoothing term (α = 0.1, β = 0.1, γ = 0.8), and **d** an acceptable parameter set (α = 0.2, β = 0.5, γ = 0.3)

Figure 5 shows the intermediate segmentation results, showing the change in behavior according to different combinations of speed parameters for two patients. First, the first through the third columns show a clear case (Patient 1). Second, the fourth through the sixth columns shows a more heterogeneous case (Patient 2) than the first case.

As shown in the first row in Fig. 5, a parameter set with strong weight for the edge term (α = 0.8, β = 0.1, γ = 0.1) restricted the expansion of boundary contour evolution, resulting in under-segmentation, particularly in Patient 2; highly irregular jags of the heterogeneous area are shown in Fig. 5(a). A larger weight for the region energy (α = 0.1, β = 0.8, γ = 0.1) produced clear boundary curves in Patient 1, but irregular isolated regions were present in Patient 2 (Fig. 5(b)). On the other hand, a larger weight for the smoothing energy (α = 0.1, β = 0.1, γ = 0.8) controlled the smoothness of the segmented tumor boundary but resulted in under-segmentation (Fig. 5(c)). We found an optimal set of parameters from a combinatorial search using visual validation as an evaluation measure, which produced acceptable segmentation results in our dataset. As a result, we determined a set of optimal parameters as shown in Fig. 5(d).

**Fig. 6:**
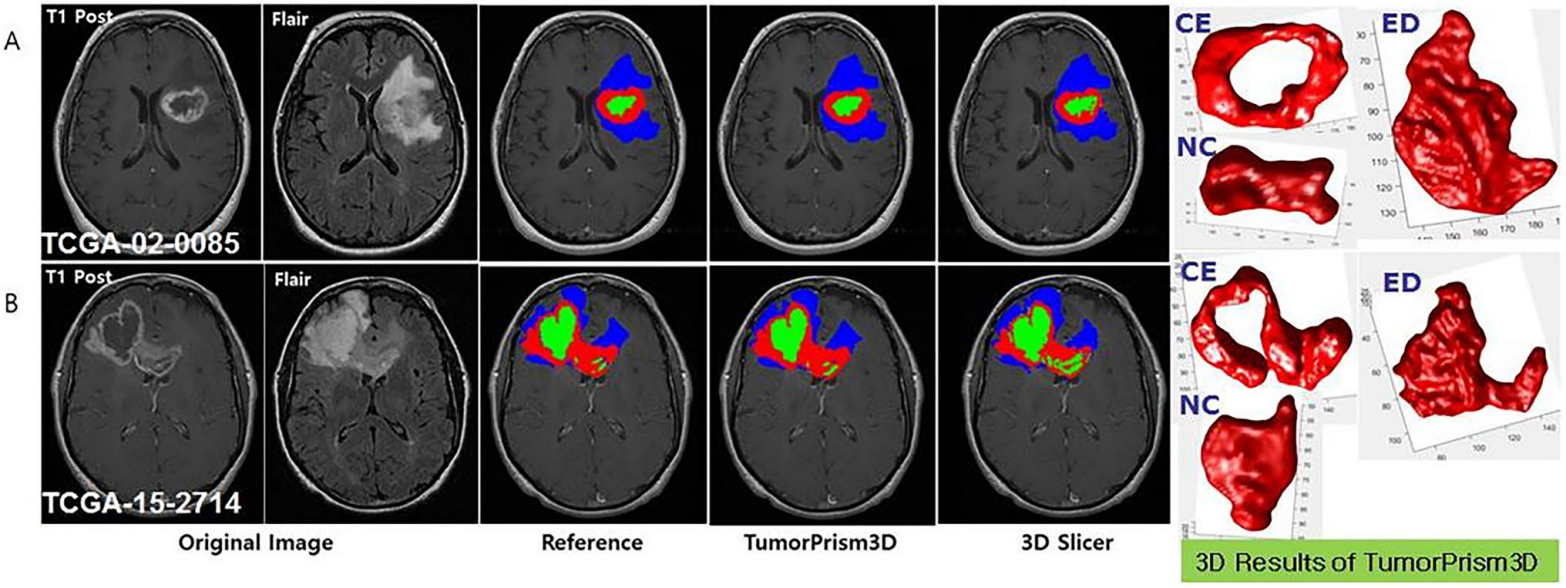
Comparison of segmentation results with two semi-automated tools (red color: contrast enhanced, green color: necrotic, blue color: edema). **A** TCGA-02-0085 **B** TCGA-15-2714

In addition, we compared the segmentation results of TumorPrism3D with those of 3DSlicer [5] as shown in Fig. 6. Figure 6A shows a case with similar results, while sample 6B shows a case with slightly different results.

To evaluate the accuracy of the segmented tumors obtained with the two semi-automated tumor segmentation tools, the DSC was computed. As shown in Table 2, TumorPrism3D achieved a better DSC than 3DSlicer (0.83 to 0.91 versus 0.8 to 0.84), with no statistically significant difference (P > 0.05).

**Table 2:** Accuracy of segmented tumors obtained with two semi-automated tumor segmentation tools

	**SW**		
**Tumor Component**	**TumorPrism3D**	**3DSlicer**	***P*** **value**
Contrast-enhancing (CE)	0.89 ± 0.12	0.82 ± 0.13	0.0601
Necrotic (NC)	0.83 ± 0.14	0.80 ± 0.11	0.1835
Edema (ED)	0.91 ± 0.11	0.84 ± 0.12	0.0662

The average computational time of TumorPrism3D was compared with that of 3DSlicer on a PC with Intel(R) Core(TM) i7-3520 CPU 2.90 GHz with 8 GB RAM, as shown in Table 3. The processing time from the drawing of the initial contour to determining the final segmentation mask for a set of randomly selected 60 tumor cases was compared. TumorPrism3D was approximately 37.4% faster than 3DSlicer for segmenting ROIs. There was a statistically significant difference (P < 0.001) in processing time between TumorPrism3D and the 3DSlicer.

**Table 3:** Comparison of computational time for TumorPrism3D and 3DSlicer according to the tumor components (seconds)

	**SW**		
**Tumor Component**	**TumorPrism3D**	**3DSlicer**	***P* Value**
Contrast-enhancing (CE)	15.47	47.65	< .001
Necrosis (NC)	13.51	34.72	< .001
Edema (ED)	29.64	72.63	< .001

**Fig. 7:**
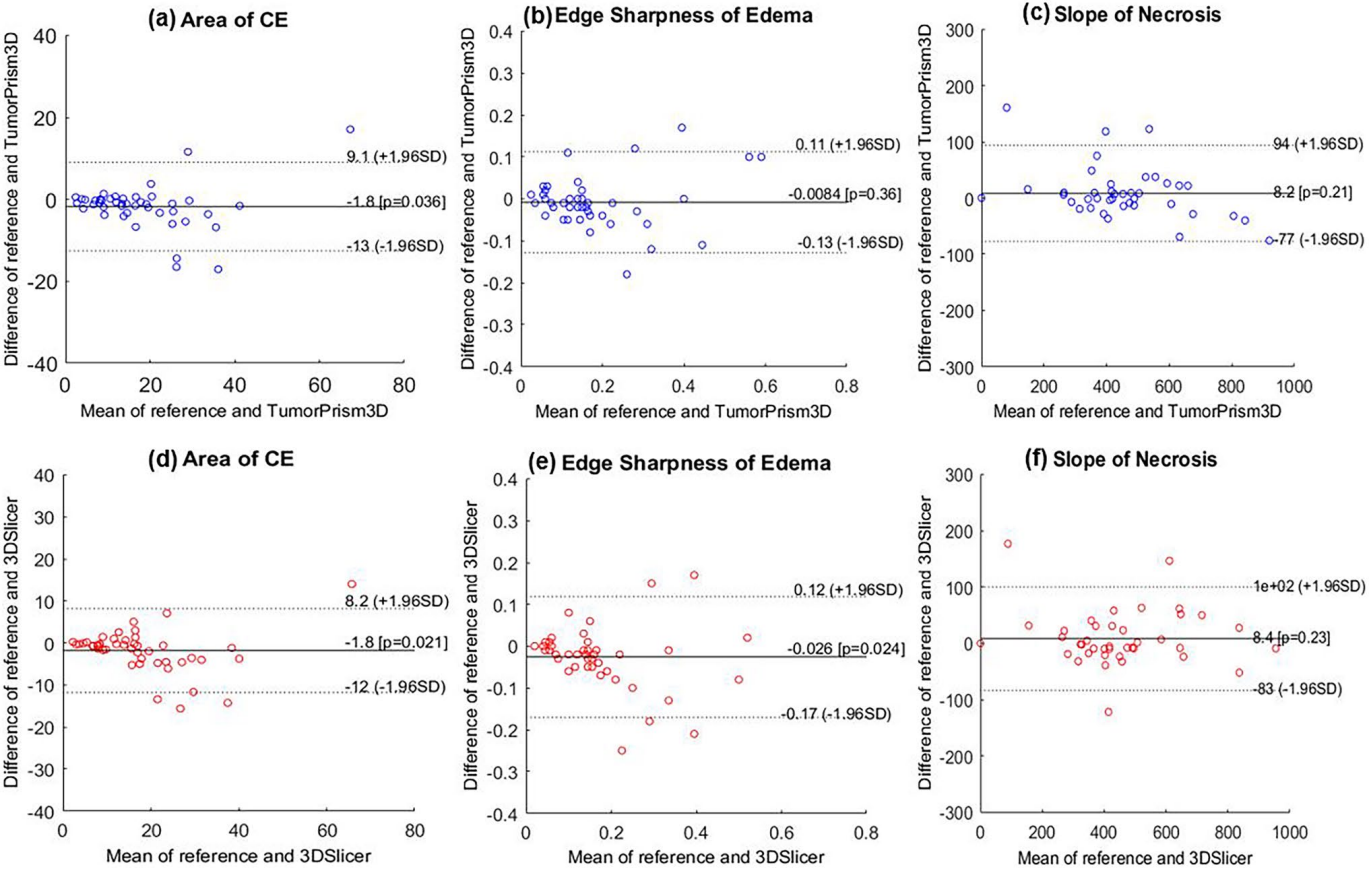
Bland-Altman plots showing the interobserver reproducibility according to the structural tumor region for CE, edema, and necrosis with TumorPrism3D (**a**–**c**) and the 3DSlicer (**d**–**f**)

Bland-Altman plot results were compared using the feature values calculated according to the two semi-automatic segmentation methods (Fig. 7). The comparative features included the area of contrast enhancement (CE), edge sharpness of edema, and slope of necrosis, and the results using the TumorPrism3D (Fig. 7(a)–(c)) are the 3DSlicer (Fig. 7(d)–(f)). TumorPrism3D showed similar or better results than 3DSlicer and the interrater agreement of the image features was acceptable.

## Discussion

Automatic segmentation of the brain tumors is a challenging task. The availability of public datasets and the well-accepted TCIA and BRATS benchmarks have recently provided a common medium for researchers to develop and objectively evaluate their methods with existing techniques. In this study, we presented TumorPrism3D, a software tool for brain tumor segmentation using MR images.

TumorPrism3D demonstrated high accuracy in segmenting all three types of tumor components in cases of glioblastoma multiforme. Comparative analysis with the widely used 3DSlicer software showed that TumorPrism3D is approximately 37.4% faster in the segmentation process from the initial contour drawing to the final segmentation mask determination. In addition, TumorPrism3D achieved a better DSC than 3DSlicer in terms of its accuracy for segmenting tumors.

In general, tumor segmentation is categorized into three methods: manual, semi-automatic, and fully automatic. Manual segmentation performed by expert radiologists is considered the gold standard to assess semi- or fully-automated tools with a subset of data. This method is labor intensive and has a rater dependency issue, and this problem further increases when the data size increases. Therefore, the use of a highly accurate semi-automated segmentation tool can be a practical option. The currently available semi-automated segmentation techniques can be categorized into point click, box draw, and sketch draw types. With any type, the user can quickly generate a 3D segmentation mask with just a simple click or draw with great efficiency. However, as the segmentation results of these methods vary depending on user input, the rater dependency issue remains; therefore, inter- and intra-rater variability must also be assessed. Several papers [17–19] have investigated the variability of tumor segmentation techniques in glioblastoma multiforme tumors using different software tools. From the results, the reliability differed depending on the software tools and tumor types studied. Segmentation is certainly a limiting factor for advancing the medical imaging process. One way to alleviate it can is to introduce a fully-automated tumor segmentation technique.

Many groups [1] have recently been developing models in this direction using ML and DL methods. In particular, DL techniques, including the convolutional neural network (CNN) model [22–26], have been demonstrated to learn representative complex features for both healthy brain tissues and tumor tissues directly from the multimodal MR images. According to published reports, fully automated segmentation techniques based on ML showed DSCs of 0.72–0.84 in edema, 0.59–0.71 in necrosis, and 0.46–0.57 in the contrast-enhanced tumor tissues on brain MR images. The Dice similarity of the segmented tumor was relatively similar to or lower than that of the semi-automated technique; in particular, the Dice similarity of the segmentation results of contrast-enhanced tumors was very low with the fully automated segmentation methods. Despite the advances in learning-based automatic segmentation methods, much research relies on interactive segmentation due to the unreliability and low specificity of fully automated methods.

In recent years, several tumor segmentation software tools/platforms for brain MR images have been introduced. In particular, 3DSlicer, which uses a grow cut model, is a comparable model to TumorPrism3D. However, the 3DSlicer tool requires additional steps and more processing time than the TumorPrism3D.

The proposed software, TumorPrism3D, is the advanced tumor segmentation tool that comprehensively addresses the routine segmentation process. It does not require specialized equipment or skills. In our experiment using two semi-automated software tools, the tumor segmentation accuracy was determined by the DSC, which ranged from 0.8 to 0.91 depending on the software tools. The two software tools employed in this study appear to provide consistently good accuracy in segmenting glioblastoma multiforme tumors compared to the high performance reported using DL [20, 21] with a segmentation accuracy of 0.77–0.88. The robustness of the software was also measured using various initial contours and was determined to be acceptable for visual assessment.

However, this software has some limitations. First, there is no direct editing function. If the segmentation result is not acceptable, the parameter settings must be re-tuned; however, the runtime is not long. Second, it is not multiparametrically segmented; only the segmentation of one tumor in a single modality can be performed. These issues will be addressed in future versions of the software. Furthermore, it is possible to merge this software into the surgery navigation system if these functions are enhanced to guide surgery.

## Conclusions

The TumorPrism3D software we developed shows promise for application in the quantitative analysis of tumor characteristics on brain MRI. In addition, TumorPrism3D demonstrated reproducible tumor segmentation performance at a fast speed. In the comparison of processing time from drawing the initial contour to determining the final segmentation mask, we showed that TumorPrism3D is approximately 37.4% faster than 3DSlicer for tumor segmentation.

In addition, segmentation of the contrast-enhancing and edema portion on brain MRI showed promising results. On the other hand, the necrotic portion showed similar or slightly better results than those obtained with 3DSlicer.

The utilization of the developed software tool in clinical applications can effectively reduce time and labor. In particular, a future version of TumorPrism3D is expected to integrate DL-based features into its segmentation model to enhance tumor segmentation performance in terms of reliability, accuracy, and user convenience.

Future research plans include upgrading the current model to a more user-friendly GUI format, and emphasizing user convenience to enable the use of TumorPrism3D in real clinical settings.

## Supplementary Information

Below is the link to the electronic supplementary material.

Supplementary file1 (DOCX 15 KB)

## Data Availability

The data used in this study can be obtained at The Cancer Imaging Archive (TCIA). Available at http://www.cancerimagingarchive.net/.
