# Supplementary material for: AI-assisted Segmentation Tool for Brain Tumor MR Image Analysis
Source: J Imaging Inform Med. 2024 Jul 8;38(1):74–83. doi: 10.1007/s10278-024-01187-7 (PMC11811333; doi:10.1007/s10278-024-01187-7)
Supplement: Supplementary file 1 — Supplementary file1 (DOCX 15 KB) [file 10278_2024_1187_MOESM1_ESM.docx]

**Supplementary 1: Three energy terms**

First, the edge energy term ($S_{e}$) drives the contour toward the tumor edges where the tumor has a distinct boundary. This term calculates the inner product of the surface normal and the gradient of an input image:

$S_{e}=sign\left( <V_{GVF,}\nabla\emptyset> \right)div\left( V_{GVF} \right)$ (1)

where $V_{GVF}$ is a gradient vector flow (GVF) field.

Second, the region energy term ($S_{r}$) is applied to define a region boundary based on a probability model for intensity distributions on target ($p_{1}$) and background ($p_{2}$) regions, assuming that each region has a distinct probability function.

$S_{r}= ({log p}_{1}(I\left( \omega\right)-log p_{2}\left( I\left( \omega\right) \right).$ (2)

where the probability of intensity values $I$ is observed at a given data partition$\omega$.

Third, the smoothing energy term ($S_{s}$) regularizes the surface curvature so as to make smooth surface, preventing irregular fluctuation caused by image noise.

$S_{s}=\left( g_{I} div\left( \frac{\nabla\emptyset}{\left| \nabla\emptyset\right|} \right)-<\nabla g,\frac{\nabla\emptyset}{|\nabla\emptyset|}> \right), g_{I}=\frac{1}{1+\left| \nabla\left( G*I \right) \right|}.$ (3)

where $g_{I}$ is a stopping term and $G*I$ is the convolution of image $I$ with the Gaussian filter $G.$

By combining three energy terms, we attempt to adapt this hybrid active surface model to reliably segment brain tumors in MR images by finding a set of weighting factors for the three energy terms tuned to the brain tumors ROIs.
